# Female Brain Work

**Published:** 1883-03

**Authors:** 


### Female Brain Work.

From the *Medical Times and Gazette* we note the following: "Referring to much that had been said and written lately about the overworking of girls and young women in schools and colleges, and that his friend Dr. Clauston had come forward as the champion of health and ignorance for women, Dr. Tuke thinks that Dr. Clauston had overstated the position of matters, that he had based his opinions more on the observation of isolated cases than on the general condition of highly educated women; that he had mistaken the wail of the one for the murmur of the many. No doubt a certain number of young women suffered and broke down whilst studying, but this did not necessarily imply that study was the cause of the breakdown. Idleness and ignorance were much more prolific causes of disease amongst women than overwork. They were the main producers of hysteria and all sorts of vapourish complaints, of many ills and evils, and of inanity, if not of insanity. As a matter of fact, it was not an easy thing to overtask the energies of the brain by work. It was not work, but worry, that killed the brain. The latter, he feared must be ever with us all. The most highly educated and hard-working woman whom he had the honor of knowing were eminently healthy. Perhaps this might be the 'survival of the fittest'; but, even granting that it was so, the more women worked the more fit women they would have. The breakdown from overstrain did occasionally take place, and the first really important symptom was sleeplessness; when that set in, there was cause for alarm. As soon as a child or young person developed continuous headache, work should be discontinued."

These are sound doctrines and deserve extensive notoriety.

